# Chronic Exposure to Low Doses of HgCl_2_ Avoids Calcium Handling Impairment in the Right Ventricle after Myocardial Infarction in Rats

**DOI:** 10.1371/journal.pone.0095639

**Published:** 2014-04-18

**Authors:** Thaís de Oliveira Faria, Gustavo Pinto Costa, Camila Cruz Pereira Almenara, Jhuli Keli Angeli, Dalton Valentim Vassallo, Ivanita Stefanon, Paula Frizera Vassallo

**Affiliations:** 1 Departamento de Ciências Fisiológicas, Universidade Federal do Espírito Santo, Vitória, Brazil; 2 Escola de Ensino Superior da Santa Casa de Misericórdia de Vitória, EMESCAM, Vitória, Brazil; Max-Delbrück Center for Molecular Medicine (MDC), Germany

## Abstract

Right ventricle systolic dysfunction is a major risk factor for death and heart failure after myocardial infarction (MI). Heavy metal exposure has been associated with the development of several cardiovascular diseases, such as MI. The aim of this study was to investigate whether chronic exposure to low doses of mercury chloride (HgCl_2_) enhances the functional deterioration of right ventricle strips after MI. Male Wistar rats were divided into four groups: Control (vehicle); HgCl_2_ (exposure during 4 weeks- 1st dose 4.6 µg/kg, subsequent dose 0.07 µg/kg/day, i.m. to cover daily loss); MI surgery induced and HgCl_2_-MI groups. One week after MI, the morphological and hemodynamic measurements and isometric tension of right ventricle strips were investigated. The chronic HgCl_2_ exposure did not worsen the injury compared with MI alone in the morphological or hemodynamic parameters evaluated. At basal conditions, despite similar maximum isometric force at L-max, relaxation time was increased in the MI group but unaffected in the HgCl_2_-MI compared to the Control group. Impairment of the sarcoplasmic reticulum (SR) function and reduction in the sarcolemmal calcium influx were observed in MI group associated with SERCA2a reduction and increased PLB protein expression. Induction of MI in chronic HgCl_2_ exposed rats did not cause any alteration in the developed force at L-max, lusitropic function or −dF/dt except for a tendency of a reduction SR function. These findings could be partially explained by the normalization in the sarcolemmal calcium influx and the increase in NCX protein expression observed only in this group. These results suggest that chronic exposure to low doses of HgCl_2_ prevents the impaired SR function and the reduced sarcolemmal calcium influx observed in MI likely by acting on NCX, PLB and SERCA2a protein expression.

## Introduction

Myocardial Infarction (MI) is currently the leading cause of morbidity and mortality worldwide, costing the public health system millions every year [1]. The common causes of MI include hypertension [2], atherosclerosis [3], diabetes [4] and a sedentary lifestyle [5].

It is well known that chronic MI can lead to heart failure (HF) [6], [7]. Indeed, after a few hours post-MI, the ischemic process in the myocardium begins to cause deleterious effects on the contractile function of the heart [8]. In addition, in isolated heart models, acute MI impaired both the force development and the contractile index [9]. Furthermore, left myocardial dysfunction is associated with an impairment in calcium handling, suggested by a reduction in SERCA and PLB proteins and a decrease in sodium/calcium exchanger (NCX) activity [10], [11]. Importantly, right ventricle dysfunction has been considered as an independent predictor of mortality and also predicts the development of HF in patients with left ventricle dysfunction [12]. Therefore, it is very important to study the alterations in right ventricular function after acute left ventricle MI to elucidate the mechanisms involved in this process.

Currently, heavy metal exposure has also been associated with the development of cardiovascular diseases such as atherosclerosis, hypertension, coronary artery disease and MI [13], [14], [15], [16]. Additionally, acute mercury intoxication can produce pulmonary hypertension [17] and impaired vascular pulmonary function in rats [18], [19]. Moreover, chronic low doses of mercury chloride (HgCl_2_) also have an deleterious effect on coronary artery function by producing an increasing resistance to blood flow that might cause contraction and relaxation impairment under vascular overload conditions [20]. In the isolated, perfused heart, chronic exposure to mercury induces alterations in calcium handling mechanisms, such as a reduction in NCX and SERCA expression and the induction of negative inotropic effect [21].

Furthermore, there was an association between the amount of mercury in hair and the risk of MI in humans [22]. Although there is a possibility that MI could occur in individuals exposed to HgCl_2_, the underling mechanisms involved in MI and low doses of HgCl_2_ are still unknown. Thus, the aim of this study was to describe the effects of MI on the contractile function of right ventricle strips after chronic exposure to low doses of HgCl_2_.

## Materials and Methods

### Animals

Studies were performed on male Wistar rats (170–200 g). All experiments were conducted in compliance with the guidelines for biomedical research as stated by the Brazilian Societies of Experimental Biology and approved by a local ethics committee of the Federal University of Espírito Santo (084/2011 CEUA-UFES). All rats had *ad libitum* access to water and rodent chow.

### Myocardial infarction induction and experimental groups

Initially, rats were divided into two groups: Control (vehicle–saline solution, i.m) and those treated with mercury chloride for 4 weeks (1^st^ dose 4.6 µg/kg, subsequent dose 0.07 µg/kg/day, i.m to cover daily loss). As previously described, this treatment led to HgCl_2_ blood levels of approximately 8 ng/mL [20], [21], [23]. In the present study this was considered as low dose of HgCl_2_ instead of a toxic dose because our treatment attained to a blood mercury content of 8 ng/ml, that is close to the levels observed in exposed humans [24], [25]. After three weeks, another two groups were included in the study: MI animals in the presence or absence of HgCl_2_ exposure. MI injury was induced after only three weeks to ensure there was a stable blood level of HgCl_2_ as describe above [23].

MI was induced as previously reported [26]. Briefly, animals were anesthetized with ketamine (50 mg/kg, i.p) and xylazine (10 mg/kg, i.p). The thorax was opened with a thoracotomy in the fourth intercostal space, and the heart was quickly exposed. With a 6.0 suture string, the left descending coronary artery was permanently occluded at approximately 2 mm from its origin. The heart was returned and the thorax closed. Mechanical respiratory support was used in the animals that did not return spontaneously to respiratory movement. In one group of animals, a fictitious surgery was performed following the same protocol as described above except without coronary occlusion. These animals were used as a non-infarcted control.

Thus, there were four experimental groups: those animals with saline exposure and MI induction (MI) or non-infarcted control (Control), and those animals with HgCl_2_ exposure and MI (HgCl_2_-MI) or non-infarcted control HgCl_2_ exposure (HgCl_2_).

### Hemodynamic measurements and scar area

Seven days after surgery, the animals were anesthetized with ketamine (90 mg/kg; AGENER, BRAZIL) and xylazine (10 mg/kg; BAYER, BRAZIL) for hemodynamic evaluation as previously described [26]. Briefly, the right common carotid artery and jugular vein was dissected and separated from the connective tissue. A fluid-filled polyethylene catheter (P50) was inserted in the carotid artery and jugular vein. Systolic (SBP) and diastolic (DBP) blood pressure and heart rate (HR) were measured after stabilization. The catheter was then advanced into the left or right ventricle, and the systolic (LVSP), end-diastolic pressure (LVEDP), and the maximum rate of pressure raise and fall (±d*P*/dt) were obtained.

To determine the infarct size, scar tissue was carefully separated from non-infarcted myocardium, and the area was measured. Infarct area was displayed as the relative infarct area in relation to the total left ventricular area [9], [26].

### Right ventricle strips

After hemodynamic evaluation, the hearts were rapidly removed and perfused through the aortic stump as the right ventricle strips were dissected. Muscle preparations were mounted for isometric tension recording and maintained in 20 mL Krebs–Henseleit solution (in mM: NaCl 118, KCl 4.7, CaCl_2_ 1.25, KH_2_PO_4_ 1.2, MgSO_4_ 1.2, NaHCO_3_ 23 and glucose 11) at 30°C and pH = 7.4, which was continuously aerated with 95% O_2_ and 5% CO_2_. Resting tension was adjusted to produce the maximal contractile force. The twitch contraction rate was controlled by isolated rectangular pulses (10 to 15 V, 12 ms duration) through a pair of platinum electrodes. The standard stimulation rate was 0.5 Hz (steady state). Isometric force development was measured with an isometric force transducer (TSD105A, Biopac) and normalized to muscle weight (g/g). Recordings started after 45 minutes to allow the muscle to adapt to the new environmental conditions. Myocardial contractility was tested by the following protocol.

1- After stabilization, myocardial contractility was tested by measuring the basal conditions (steady state contractions). The following parameters were determined: peak isometric force, time to peak tension, relaxation time to 90%, and the +dP/dt and −dP/dt time derivatives of the right ventricular force development.

2- Post-rest potentiation was used to provide indirect information about the function of the sarcoplasmic reticulum (SR). Post-rest potentiation depends on the pause duration and on the amount of calcium stored at intracellular sites. Pause intervals of various durations (15, 30 and 60 s) were used, and the results are presented as relative potentiation (the amplitude of post-rest potentiation divided by steady-state contractions) to normalize the data from different preparations [27].

3- The post-rest contraction was obtained after 10 min without stimulation and in the calcium-free solution containing 5 mM caffeine. To achieve post-rest contraction, the calcium-free solution was replaced with Krebs's solution (with 1.25 mM calcium) seconds before the electric stimulation. The first contraction after rest was taken as an index of the sarcolemmal calcium influx [28].

4- The contractile machinery of right ventricle strips during tetanic stimulation was evaluated. Tetanic tension was obtained after treatment with 5 mM caffeine for 20 minutes at a rate of 10 Hz for a 15 s duration as previously described [29]. The tetanic contractions developed a fast upstroke (tetanic peak force) followed by a slow decay (tetanic plateau force), and the tension was measured at the peak and in the middle of the plateau. All results were expressed as a percentage of the force of the respective steady state contractions.

5- The positive inotropic response produced by isoproterenol added to the bath (10^−4^ M), and the peak isometric force was measured. The results were expressed as a percentage of the force of the respective steady state contractions.

At the end of the experiment, the right ventricle strip was removed and weighed for force normalization (g/g).

### Western blot analyses

Western blotting was performed as previously described [20]. Proteins from homogenized right ventricles were separated by 10% or 15% SDS-PAGE gels and then transferred onto nitrocellulose membranes, which were incubated overnight with mouse monoclonal antibodies for SERCA2a (1∶1000, Thermo Scientific, IL, USA), PLB (1∶1000, Thermo Scientific, IL, USA USA), phosphorylated PLB at serine 16 (1∶5000, Badrilla, UK) and NCX (1∶1000, Thermo Scientific, IL, USA). After washing, the membranes were incubated with anti-mouse (1∶5000, Stressgen, Victoria, Canada) immunoglobulin antibodies conjugated to horseradish peroxidase for 1 hour. After thorough washing, immunocomplexes were detected using an enhanced horseradish peroxidase/luminal chemiluminescence system (ECL, Amersham International, Little Chalfont, UK) and film (Hyperfilm ECL International). Signals on the immunoblot were quantified using the Image J computer program. Each membrane was reprobed to determine GAPDH expression using a mouse monoclonal antibody (1∶5000, Abcam Cambridge, MA, USA).

### Data analysis and Statistics

All values are expressed as the mean ± S.E.M with “n” indicating the number of observations. The results were analyzed using randomized Student's t-tests and one-way ANOVAs. When the ANOVAs showed a significant treatment effect, Tukey's *post hoc* tests were used to compare individual means. Differences were considered statistically significant at p<0.05. The data were analyzed and the figures were plotted with GraphPad Prism™ (version 5.0, GraphPad Software, USA). For protein expression, the data were expressed as the signal ratio between the protein of interest and GAPDH.

## Results

### Chronic exposure to HgCl_2_ did not worsened the morphological or hemodynamic parameters related to infarction

Chronic exposure to HgCl_2_ alone did not change the overall morphological and hemodynamic measurements evaluated when compared to Control group (p>0.05) Because these results are similar to the control group, they will not be included in the body of results but presented as supporting information (Table S1). General morphological and hemodynamic characteristics of the Control, MI and HgCl_2_-MI groups are depicted in Table 1. Body weight (BW) gain was observed in all groups between the 1^st^ and 3^rd^ week. At the 4^th^ week, the BW of the control rats continued to increase, while BW loss was evident in the animals that underwent MI surgery (p<0.05, MI and HgCl_2_-MI). Additionally, the relative weight of right ventricle (RV/BW) and the relative weight of lungs (LW/BW) were increased in both groups of MI when compared with the Control group (p<0.05). The same scar size was observed in MI and HgCl_2_-MI groups (p>0.05) (Table 1).

**Table 1:** Morphological and hemodynamic parameters.

Groups	Control	MI	HgCl_2_-MI
**Body Weight (g)**			
***Baseline***	191.3±3.6	189.1±3.6	191.1±5.4
***3 week***	301.5±5.8	301.0±6.8	300.3±6.3
***4 week***	334.5±7.9	289.7±5.9*	286.5±8.0*
**LV/BW (mg/g)**	2.00±0.07	2.03±0.08	2.14±0.07
**RV/BW (mg/g)**	0.50±0.02	0.66±0.04*	0.66±0.04*
**LW/BW (mg/g)**	4.89±0.14	7.38±0.63*	7.18±0.60*
**Scar size (%)**		35.7±2.0	37.1±3.3
**HR (BPM)**	306.0±27.5	240.0±12.6*	240.0±6.3*
**SBP (mmHg)**	114.7±3.6	94.7±2.7*	93.9±1.5*
**DBP (mmHg)**	88.5±3.6	71.8±1.7*	70.2±3.6*
**LVSP (mmHg)**	133.9±3.8	106.3±3.9*	103.4±2.7*
**LVEDP (mmHg)**	4.7±0,6	16.7±1.6*	16.6±1.6*
**LV +dP/dt (mmHg/s)**	6816±191	3739±138*	3613±104*
**LV −dP/dt (mmHg/s)**	−6644±304	−3962±163*	−3779±108*
**RVSP (mmHg)**	31.2±2.1	31.6±1.2	31.5±1.8
**RVEDP (mmHg)**	2.04±0.24	5.14±0.54*	4.40±0.48*
**RV +dP/dt (mmHg/s)**	1470.0±114.0	964.7±103.3*	1034±118.9*
**RV −dP/dt (mmHg/s)**	−1438.1±86.8	−1134.2±6.6*	−1127.0±65.8*

Body weight, relative weight of left (LV/BW) and right ventricle (RV/BW), relative weight of lungs (LW/BW), scar size, systolic (SBP) and diastolic (DBP) blood pressure, heart rate (HR), left (LVSP) and right (RVSP) ventricle systolic pressure, left (LVEDP) and right (RVEDP) end diastolic pressure, positive (+dP/dt) and negative (−dP/dt) rates of pressure development in left (LV) and right (RV) ventricle of Control, infarcted (MI) and continuous exposure to HgCl_2_ - infarcted animals (HgCl_2_-MI). Results are reported as means ± SEM for 8–10 animals.

*p<0.05 compared to Control (one-way repeated measures ANOVA followed by the Tukey test).

The hemodynamic function was impaired in both groups that underwent MI, which was compatible with the development of cardiac failure. Hemodynamic parameters, such as systolic (SBP) and diastolic (DBP) blood pressure, and left (LVSP) systolic ventricle pressure, positive (+dP/dt) and negative (−dP/dt) rates of pressure development in left (LV) and right (RV) ventricles, were reduced in the MI and HgCl_2_-MI groups when compared with the Control group. Moreover, the left and right end diastolic pressures (LVEDP and RVEDP) of the MI and HgCl_2_-MI groups were significantly higher than the Control group (Table 1). In brief, chronic HgCl_2_ exposure did not result in more alterations in the morphological or hemodynamic parameters than what was already caused by infarction.

### HgCl_2_ chronic exposure did not affected the overall myocardial contractility of the right ventricle in infarcted animals in basal conditions

**Figure 1:**
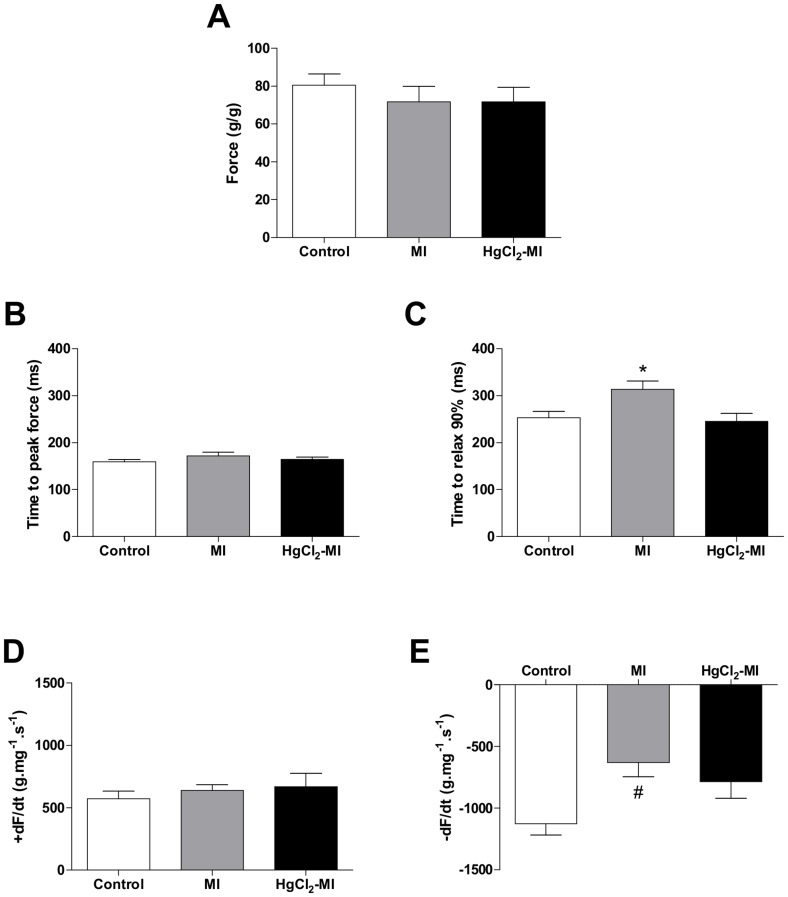
Basal measurements from the right ventricle strips. The effect of myocardial infarction in rats with continuous exposure to HgCl_2_ on the basal conditions of isometric force (A), time to peak tension (B), relaxation time to 90% (C), +dF/dt (D) and −dF/dt (E) of rat right ventricular strips from the Control, MI and HgCl_2_-MI groups. The results are reported as the means ± SEM for 10–12 animals per group. MI = myocardial infarction; dF/dt = time derivatives of right ventricular force development. *P<0.05 *vs* Control and HgCl_2_-MI; # P<0.05 *vs* Control (one-way ANOVA followed by Tukey's *post hoc* tests).

The continuous exposure to HgCl_2_ alone did not change the overall myocardial contractility of the right ventricle strips when compared to the Control group (p>0.05). Because these results are similar to the control group, they will not be included in the body of results but presented as supporting information (Figures S1, S2, S3, S4, S5). Figure 1 illustrates the effects of MI and HgCl_2_ exposure in basal conditions from the right ventricle strips of Control, MI and HgCl_2_-MI exposed groups. MI did not affect the isometric force developed (Control 80.4±6.0; MI 71.7±8.1 and HgCl_2_-MI 71.7±7.6 g/g, p>0.05), time spent to peak force (Control 159±5.1; MI 172±7.8 and HgCl_2_-MI 164±4.5 ms, p>0.05) and +dF/dt (Control 574±57.7; MI 639±45.5 and HgCl_2_-MI 669±109 g.mg^−1^.s^−1^, p>0.05) in all groups evaluated. However, an increase in the relaxation time (to 90%) (MI: 313±17.7 vs Control: 253±13.1 and HgCl_2_-MI: 245±17.1 ms, p<0.05) associated with impairment in −dP/dt (MI: −630±115.4 vs Control: −1129±90.2 and HgCl_2_-MI:−835±141.4 g.mg^−1^.s^−1^, p<0.05) was observed only in the MI group. These results suggest that continuous exposure to HgCl_2_ plus MI caused less damage to the contractility function of the right ventricle strips when compared with MI alone.

### Chronic exposure to HgCl_2_ avoids the impaired SR function and the reduced sarcolemmal calcium influx caused by infarction

**Figure 2:**
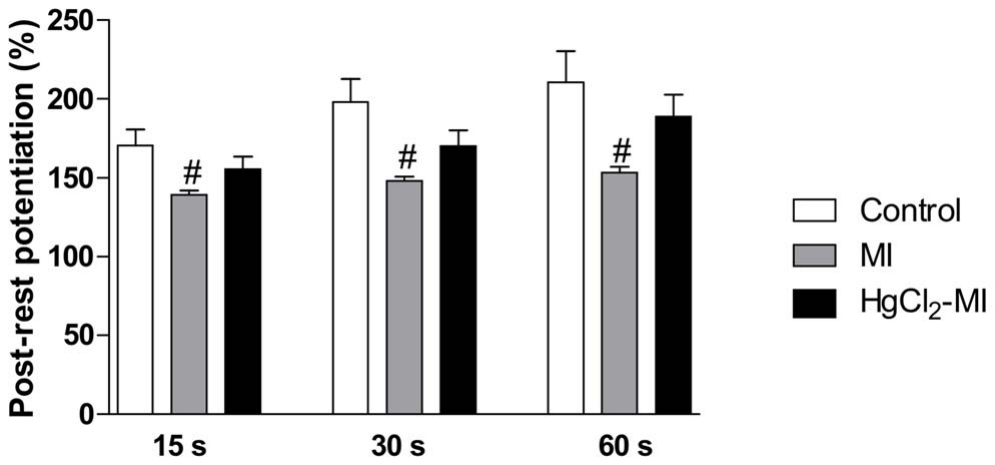
Functional assessment of sarcoplasmic reticulum. The effect of myocardial infarction in rats with continuous exposure to HgCl_2_ on relative potentiation (ratio of post-rest contractions and steady-state contractions) of isometric contractions obtained after a 15, 30, and 60 s pause in rat right ventricular strips from the Control, MI and HgCl_2_-MI groups. The results are reported as the means ± SEM for 10–12 animals per group. MI = myocardial infarction. # P<0.05 *vs* Control in the same time pause (one-way ANOVA followed by Tukey's *post hoc* tests).

**Figure 3:**
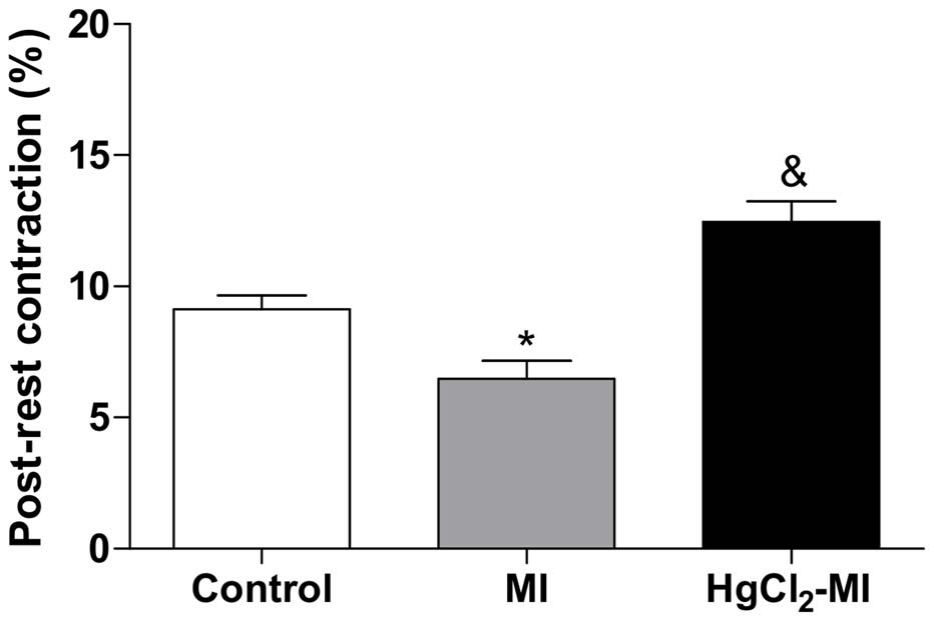
Measurements of sarcolemmal calcium influx. The effect of myocardial infarction in rats with continuous exposure to HgCl_2_ on relative potentiation (ratio of post-rest contraction and steady-state contractions) of isometric contractions obtained after a 15 min pause in rat right ventricular strips from the Control, MI and HgCl_2_-MI groups. The results are reported as the means ± SEM for 10–12 animals per group. MI = myocardial infarction.*P<0.05 *vs* Control and HgCl_2_-MI; & P<0.05 *vs* Control and MI (one-way ANOVA followed by Tukey's *post hoc* tests).

To investigate the putative role of the SR and sarcolemmal calcium influx, two protocols were implemented: the post-rest potentiation and the post-rest contraction. In the first protocol, after all of the stimuli pauses, MI decreased the relative potentiation (15 s: 139±2.8, 30 s: 148±2.9, 60 s: 153±3.7%) when compared with the Control group (15 s: 171±10, 30 s: 198±14.6, 60 s: 211±19.8%, p<0.05),. However, no differences were observed in the HgCl_2_-MI animals (15 s: 156±8.1, 30 s: 170±10, 60 s: 189±13.8% p>0.05) when compared to the Control or MI animals (Figure 2), suggesting that the SR function was the most impaired in the MI group compared to the HgCl_2_-MI group. Furthermore, in the second protocol, the animals with a continuous exposure to HgCl_2_ plus MI (12.4±0.8%, p<0.05) had a post-rest contraction increase by approximately 36% when compared with the Control rats (9.13±0.7%) and by 93% when compared to the MI rats (6.45±0.8%) (Figure 3). In contrast, the post-rest contraction was impaired in the MI group when compared with the Control group. These results suggest that there is a reduction in the sarcolemmal calcium influx in the MI group but not in HgCl_2_-MI group.

### Chronic exposure to HgCl_2_ did not affect tetanic contractions in infarcted animals

We also investigated whether the continuous exposure to HgCl_2_ following MI affected the contractile machinery by measuring tetanic contractions. To do so, tetanic contractions were obtained after the blockade of the SR activity by caffeine (5 mM during 30 minutes). In the absence of calcium from the SR to activate contraction, the tetanic contractions became dependent on the sarcolemmal calcium influx and on the myofilaments calcium sensitivity.

**Figure 4:**
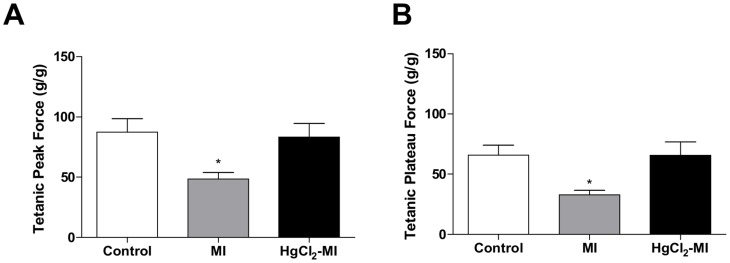
Tetanic contractions. The effect of myocardial infarction in rats with continuous exposure to HgCl_2_ on relative tetanic peak force (A) and relative tetanic plateau force (B) in rat right ventricular strips from the Control, MI and HgCl_2_-MI groups. The results are reported as the means ± SEM for 10–12 animals per group. MI = myocardial infarction; * P<0.05 *vs* Control and HgCl_2_-MI (one-way ANOVA followed by the Tukey's *post hoc* test).

Figure 4 illustrates that MI depressed the tetanic contractions developed by the right ventricle strips at tetanic peak force (Control 87.3±11.3; MI 48.5±5.5 and HgCl_2_-MI 83.2±11.4 g/g, p<0.05) and at the middle of the force decay (plateau force) (Control 65.9±8.2; MI 32.9±3.6 and HgCl_2_-MI 65.6±11.3 g/g, p<0.05). However, tetanic contractions were not affected in animals that underwent HgCl_2_ exposure plus MI. Again, these findings suggests that HgCl_2_ exposure in infarcted animals affected the myofilament calcium sensitivity of right ventricle strips less than infarction alone.

### Inotropic response to β-adrenergic stimuli was equally reduced in infarcted animals independent of chronic exposure to HgCl_2_

**Figure 5:**
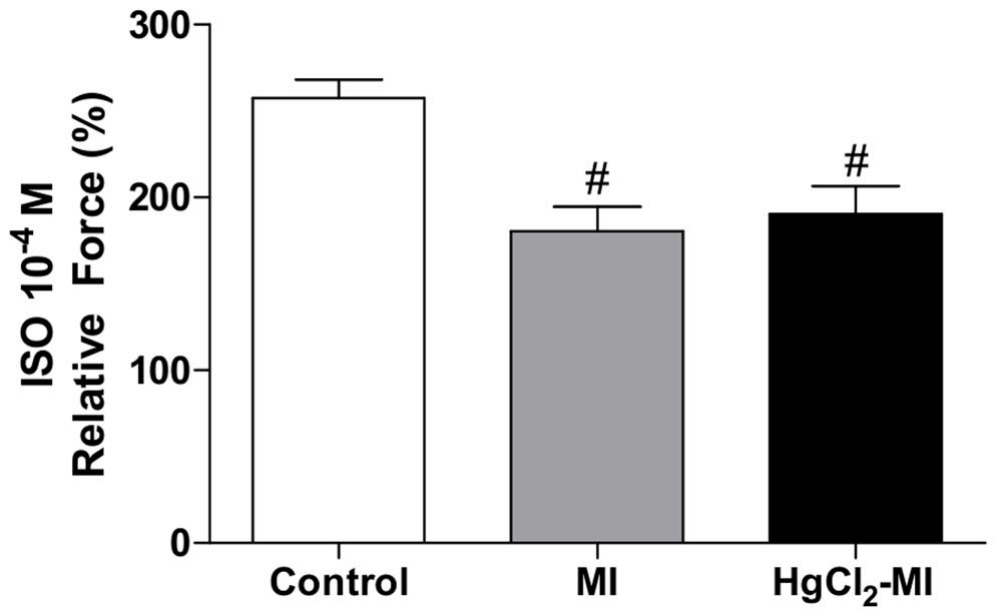
Response to β-adrenergic stimuli. The effect of myocardial infarction in rats with continuous exposure to HgCl_2_ on isoproterenol (ISO, 10^−4^ M) conditions compared to steady-state condition of isometric force. The results are reported as the means ± SEM for 10–12 animals per group. MI = myocardial infarction; # P<0.05 *vs* Control (one-way repeated measures ANOVA followed by the Tukey's *post hoc* test).

Isoproterenol was used to test whether MI in animals exposed to HgCl_2_ could maintain the myocardial response to inotropic interventions. Isoproterenol administration (10^−4^ M) induced the relative force by a smaller increment (Control 257±13.6; MI 184±8.9 and HgCl_2_-MI 174±8.9%, p<0.05) in both groups that underwent MI (MI and HgCl_2_-MI) compared with the Control group (Figure 5).

### Calcium handling proteins were differentially expressed in infarcted animals with chronic exposure to HgCl_2_

**Figure 6:**
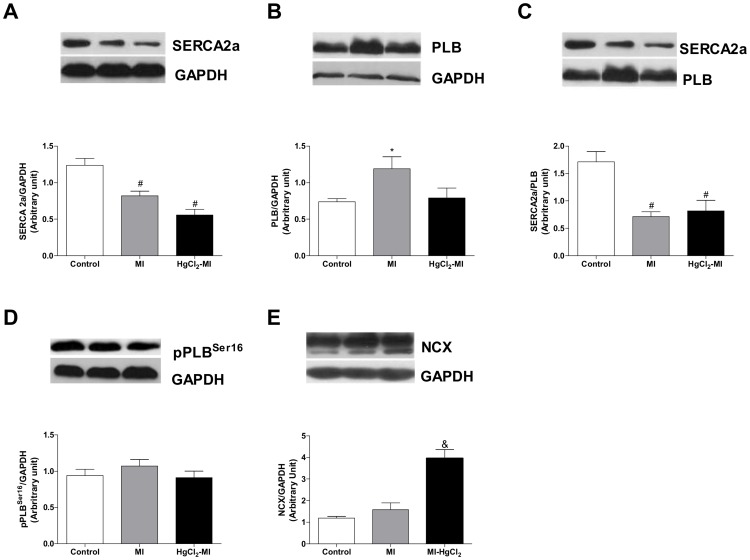
Right ventricle proteins levels. The effect of myocardial infarction in rats with continuous exposure to HgCl_2_ on the densitometry analysis of the Western blot for SERCA2a (A), PLB (B), PLB phosphorylated at serine 16 (C), SERCA/PLB ratio (D), NCX (E) in rat right ventricular strips from Control, MI and HgCl_2_-MI groups. The results are reported as the means ± SEM for 5–6 animals per group. MI = myocardial infarction; * P<0.05 *vs* Control and HgCl_2_-MI; # P<0.05 *vs* Control; & P<0.05 *vs* Control and MI (one-way ANOVA followed by the Tukey's *post hoc* test).

To evaluate the calcium handling mechanisms involved in the enhanced performance of the right ventricle strips from HgCl_2_-MI, the expression of NCX, SERCA2a, phospholamban and its phosphorylated fraction proteins were measured. The continuous exposure to HgCl_2_ alone reduced SERCA and pPLB^Ser16^ expression of right ventricle tissue when compared to the Control group (p<0.05), while the others proteins did not change (Figure S6). SERCA2a expression was reduced in both infarcted groups, while phospholamban expression was increased only in the MI group. These changes led to a reduction in SERCA/PLB ratio in the MI and HgCl_2_-MI groups, suggesting that there was a reduction in calcium uptake by the SR after MI surgery. The pPLB^Ser16^ did not change in any of the evaluated groups. Moreover, the expression of NCX was increased only in the HgCl_2_-MI group (Figure 6). These results highlight the gain in calcium homeostasis in the right ventricle of HgCl_2_-MI.

## Discussion

Our results suggest that chronic exposure to HgCl_2_ plus MI caused less damage to the contractile function of the right ventricle strips when compared with MI alone, and this might be associated with an increase in sarcolemmal calcium influx. Moreover, in accordance with the functional results, the present study showed an increase in NCX protein levels and a reduction in SERCA2a protein levels in the right ventricles of animals that underwent chronic HgCl_2_ exposure and acute MI.

Cardiac dysfunction is commonly characterized by alterations in lusitropism and inotropism associated with impaired in SR function, sarcolemmal calcium influx and damage in calcium regulatory proteins [8], [9]. Recent reports have shown that the events of MI were related to chronic exposure to mercury [13], [14]. However, to the best of our knowledge, this is the first report describing the underlying mechanisms involved in the association between early MI and chronic exposure to HgCl_2_ in right ventricle strips using a rat model.

It is well established that after an acute MI, the heart initiates a complex series of changes to preserve cardiac function, known as ventricular remodeling [30]. The underlying mechanism of ventricular remodeling is not completely elucidated, but it is known that it involves hypertrophy of cardiomyocytes, apoptosis and fibrosis of the myocardial matrix [8]. The morphological and hemodynamic data in this experiment illustrated that the left and right ventricle function was impaired in the MI rats (MI and HgCl_2_-MI groups), which is consistent with the findings of Faria et al. (2011). In addition, body weight loss observed post-MI suggests high severity in the progress of ventricular remodeling [26]. As expected, the MI groups showed an increased LVEDP [9], [26], [31], which consequently triggered right ventricle hypertrophy as well as increased RVEDP [32].

However, our results show that chronic HgCl_2_ exposure did not add more injury than MI induced alone. Consequently, the same alterations found in MI group were observed in HgCl_2_-MI group. Indeed, it was already reported that exposure to low concentrations of HgCl_2_ does not modify arterial blood pressure and left ventricle pressure except for a small increase in LVEDP in rats [21]. In reality, the consequences of acute MI and continuous exposure to mercury are still unclear. In a recent study among US health professionals, no association was found between mercury levels in nails and risk of coronary heart disease, stroke or cardiovascular disease [33]. However, Guallar et al. (2002) have shown an independent and graded association between mercury levels in toenails and the risk of MI in an important case-control study [34]. In addition, another report found a significant relationship between hair mercury, 24-hour urine mercury and cardiovascular events even in subtoxic amounts [22].

Anavekar et al. (2008) showed that RV systolic dysfunction is a major risk factor for death, sudden death, heart failure, and stroke after MI [35]. However, despite broad evidence that right ventricular function is a critical determinant of the clinical response to a variety of cardiovascular diseases, there is still limited knowledge in distinguishing physiologic properties of the RV under normal circumstances and in response to pathologic insults [11]. In the current study, we observed that in the right ventricle strips from rats that underwent controlled chronic exposure to HgCl_2_ there were no changes in any of the contractility parameters evaluated. However, our focus was to study the association between chronic exposure to small doses of HgCl_2_ with acute MI.

The present study demonstrated that MI after chronic HgCl_2_ exposure did not modify basal contraction parameters in rats, such as peak isometric force, time to peak tension, relaxation time to 90%, and the +dP/dt and −dP/dt of right ventricular force development. Additionally, our results suggest that the function of the SR is maintained, at least in part, in isolated right ventricle strips of HgCl_2_-MI animals when compared with the Control group. Our findings show that a possible explanation for the maintenance of similar force is the increase in calcium influx observed when comparing post-rest contractions responses between the groups. Another possibility we investigated was the behavior of the tetanic tension, which occurred after the blockade of SR activity by caffeine. In such conditions, the contractions become dependent on the sarcolemmal calcium influx and on the myofilament calcium sensitivity. The sarcolemmal calcium influx was increased and the tetanic tension was normalized, which suggests that there was an increase in myofilament sensitivity in the HgCl_2_-MI group.

Certainly, HgCl_2_ is not protective against MI; in contrast, as noted above, there are indications that mercury causes cardiovascular disease [13], [21], [22]. At higher concentrations, mercury reduces tetanic tension development, myosin ATPase activity and induces a negative inotropism in the left ventricle [36], [37]. Additionally, low mercury concentrations produce a negative inotropic effect and reduces β-adrenergic response in perfused hearts. However, our results suggest that in infarcted animals, chronic exposure to low doses of HgCl_2_ avoids the impaired lusitropic function (−dF/dt), SR function, reduced sarcolemmal calcium influx and lowered myofilament sensitivity to calcium.

The underlying mechanisms that could explain our findings might be related to alterations in NCX, SERCA and PLB protein levels or function [37]. Previous experiments have shown that HgCl_2_ exposure reduces SERCA and NCX protein expression and increases PLB and pPLB^Ser16^ protein expression in whole cardiac tissue [21]. Our findings showed partial similarity because SERCA and pPLB^Ser16^ expression were also reduced, while the others proteins did not change. However, our results were obtained from right ventricle samples, instead of whole heart. These differences might explain why PLB and NCX expression did not change. The possible mechanisms occurs when mercury enters into the cells and binds to SH groups, affecting protein activity [38] and DNA by direct action on the microtubules [39]. Otherwise, the mechanical activity in the right ventricle strips was preserved even with underwent HgCl_2_ exposure.

Multiple studies suggest that SR calcium uptake is impaired in the failing human heart, which is an outcome that is attributable to several mechanisms such as the reduced expression and function of SERCA2a and the increased inhibitory activity of PLB [6], [38]. In the MI group, a reduction in SERCA2a expression and an increase in PLB protein levels was observed as expected. Taken together, these results may suggest that the impairment of the SR function causes a delay in SR calcium reuptake and consequently impairs lusitropic function, which might be associated with an impaired contractility index (−dF/dt). However, continuous exposure to HgCl_2_ plus MI also reduced SERCA expression but did not modified PLB expression. These findings corroborated our functional results that showed a reduced impairment in the SR function and normal lusitropic function when compared to MI alone. Moreover, the high NCX levels observed only in the animals with continuous exposure to HgCl_2_ plus MI might explain the normal relaxing time observed even in the presence of more sarcolemmal calcium influx and normal lusitropic and −dF/dt parameters.

A failing myocardium is marked by spontaneous diastolic SR calcium release, which leads to spontaneous and highly variable diastolic sarcomere contractions and contributes to the reduction of inotropic effects in HF [40]. In accordance with these characteristics, we observed reduced β-adrenergic function in both MI groups (MI and HgCl_2_-MI), which was reflected by a reduction in the inotropic response after beta-adrenergic stimuli.

In conclusion, our results indicate that chronic exposure to low doses of HgCl_2_ avoids the impaired SR function and the reduced sarcolemmal calcium influx observed in MI, likely by acting on PLB, SERCA2a and NCX protein expression.

## Supporting Information

Figure S1 **Basal measurements from right ventricle strips.** The effect of HgCl_2_ exposure on basal conditions of isometric force (A), time to peak tension (B), relaxation time to 90% (C), +dF/dt (D) and −dF/dt (E) of rat right ventricular strips from the Control and HgCl_2_ groups. The results are reported as the means ± SEM for 8–10 animals per group. dF/dt = time derivatives of right ventricular force development.
(TIF)

Figure S2 **Functional assessment of sarcoplasmic reticulum.** The effect of HgCl_2_ exposure on relative potentiation (ratio of post-rest contractions and steady-state contractions) of isometric contractions obtained after a 15, 30 and 60 s pauses in rat right ventricular strips from the Control and HgCl_2_ groups. The results are reported as the means ± SEM for 8–10 animals per group.
(TIF)

Figure S3 **Measurements of sarcolemmal calcium influx.** The effect of HgCl_2_ exposure on relative potentiation (ratio of post-rest contraction and steady-state contractions) of isometric contractions obtained after a 15 min pause in rat right ventricular strips from the Control and HgCl_2_ groups. The results are reported as the means ± SEM for 8–10 animals per group.
(TIF)

Figure S4 **Tetanic contractions.** The effect of HgCl_2_ exposure on relative tetanic peak force (A) and relative tetanic plateau force (B) in rat right ventricular strips from the Control and HgCl_2_ groups. The results are reported as the means ± SEM for 8–10 animals per group.
(TIF)

Figure S5 **Response to β-adrenergic stimuli.** Effects of HgCl_2_ exposure in rats on isoproterenol (ISO, 10^−4^ M) conditions compared to steady-state condition of isometric force in rat right ventricular strips from the Control and HgCl_2_ groups. The results are reported as the means ± SEM for 8–10 animals per group.
(TIF)

Figure S6 **Right ventricle proteins levels.** Effects of HgCl_2_ exposure in rats on the densitometry analysis of the Western blot for SERCA2a (A), PLB (B), PLB phosphorylated at serine 16 (C), SERCA/PLB ratio (D), NCX (E) in rat right ventricular strips from Control and HgCl_2_ groups. The results are reported as the means ± SEM for 5–6 animals per group. * P<0.05 *vs* Control (Student t test).
(TIF)

Table S1 **Morphological and hemodynamic parameters.**
(DOC)
