# Supplementary material for: Chronic Exposure to Low Doses of HgCl2 Avoids Calcium Handling Impairment in the Right Ventricle after Myocardial Infarction in Rats
Source: PLoS One. 2014 Apr 18;9(4):e95639. doi: 10.1371/journal.pone.0095639 (PMC3991698; doi:10.1371/journal.pone.0095639)
Supplement: Table S1 — Morphological and hemodynamic parameters. (DOC) [file pone.0095639.s007.doc]

**Supporting Information Table**

**Table S1. Morphological and hemodynamic parameters.**

|  | **Control** | **HgCl_2_** |
| --- | --- | --- |
| **Body Weight (g)** |  |  |
| ***Baseline*** | 191.3±3.6 | 187.1±2.7 |
| ***3 week*** | 301.5±5.8 | 301.0±4.7 |
| ***4 week*** | 334.5±7.9 | 332.8±6.6 |
| **LV/BW (mg/g)** | 2.00±0.07 | 2.02±0.05 |
| **RV/BW (mg/g)** | 0.50±0.02 | 0.50±0.02 |
| **LW/BW (mg/g)** | 4.89±0.14 | 4.93±0.23 |
| **FC (BPM)** | 306.0±27.5 | 292.0±12.2 |
| **SBP (mmHg)** | 114.7±3.6 | 112.5±3.9 |
| **DBP (mmHg)** | 88.5±3.6 | 84.5±4.2 |
| **LVSP (mmHg)** | 133.9±3.8 | 132.8±3.1 |
| **LVEDP (mmHg)** | 4.7±0,6 | 5.6±0.9 |
| **LV +dP/dt (mmHg/s)** | 6816±191 | 6957±255 |
| **LV - dP/dt (mmHg/s)** | -6644±304 | -6586±100 |
| **RVSP (mmHg)** | 31.2±2.1 | 30.7±1.4 |
| **RVEDP (mmHg)** | 2.04±0.24 | 2.56±0.34 |
| **RV +dP/dt (mmHg/s)** | 1470.0±114.0 | 1279.8±44.2 |
| **RV - dP/dt (mmHg/s)** | -1438.1±86.8 | -1305.2±80.3 |

Body weight, relative weight of left (LV/BW) and right ventricle (RV/BW), relative weight of lungs (LW/BW), scar size, systolic (SBP) and diastolic (DBP) blood pressure, heart rate (HR), left (LVSP) and right (RVSP) ventricle systolic pressure, left (LVEDP) and right (RVEDP) end diastolic pressure , positive (+dP/dt) and negative (-dP/dt) rates of pressure development in left (LV) and right (RV) ventricle of Control and HgCl_2_ groups. Results are reported as means ± SEM for 7-10 animals.
